# Default mode network alterations during implicit emotional faces processing in first-episode, treatment-naive major depression patients

**DOI:** 10.3389/fpsyg.2015.01198

**Published:** 2015-08-12

**Authors:** Huqing Shi, Xiang Wang, Jinyao Yi, Xiongzhao Zhu, Xiaocui Zhang, Juan Yang, Shuqiao Yao

**Affiliations:** ^1^Medical Psychological Institute, Second Xiangya Hospital, Central South University, Changsha, China; ^2^Department of Psychology, Shanghai Normal University, Shanghai, China; ^3^National Technology Institute of Psychiatry, Central South University, Changsha, China; ^4^Department of Psychology, Hainan Medical College, Haikou, China

**Keywords:** major depressive disorder, emotional faces processing, functional magnetic resonance imaging, default mode network, independent component analysis

## Abstract

Previous studies have focused on resting-state default mode network (DMN) alterations in the development and maintenance of depression; however, only a few studies have addressed DMN changes during task-related processing and their results are inconsistent. Therefore, we explored DMN patterns in young adult patients with first-episode, treatment-naïve major depressive disorder (MDD) performing an implicit emotional processing task. Patients with MDD (*N* = 29) and healthy controls (*N* = 33) were subjected to functional magnetic resonance imaging (fMRI) at rest and while performing a gender judgment task. Group independent component analysis (ICA) was used to identify DMN component under task state for both groups. The DMN of participants with MDD had decreased functional connectivity in bilateral prefrontal areas compared to controls. Right prefrontal gyrus connectivity for MDD patients correlated negatively with scores on maladaptive scales of the Cognitive Emotion Regulation Questionnaire (CERQ). Our findings suggest that depressed people have altered DMN patterns during implicit emotional processing, which might be related to impaired internal monitoring and emotional regulation ability.

## Introduction

The examination of resting-state brain activity through fMRI has become an important area of neuroscientific study. The default mode network (DMN), first found by Raichle et al. (2001) in subjects at rest, refers to a network of brain regions with spontaneously organized brain activities, namely the posterior cingulate cortex (PCC), precuneus (PCu), inferior parietal lobule, ventral anterior cingulate cortex (ACC), and ventral prefrontal cortex (PFC). Researchers have speculated that the DMN could be related to external environment monitoring and self-consciousness maintenance (Gusnard et al., 2001), spontaneous thinking production (Mason et al., 2007), self-related emotion, memory, and cognition (Cabeza et al., 2002). Researchers have observed consistent deactivation of DMN brain areas when subjects shift from a resting phase to an on-task phase across different tasks (Shulman et al., 1997; McKiernan et al., 2006). Since these DMN areas showed task-independent negative activation in attention-obliging or goal-directed tasks, these brain areas have also been called a task-negative network, which anti-correlates with task-induced brain activation areas referred to as a task-positive network (Fox et al., 2005).

Previous studies of major depressive disorder (MDD-associated DMN alterations have been inconsistent). Some researchers found that, compared with healthy controls, depressed patients showed increased resting state functional connectivity of the DMN areas, such as the subgenual cingulate cortex and thalamus, especially increased medial PFC (mPFC) functional connectivity with other resting-state brain networks, including the cognitive control network, affective network, and DMN itself (Greicius et al., 2007; Sheline et al., 2010). Other researchers found that part of the DMN areas showed decreased resting state functional connectivity, such as the PCC/PCu and caudate, cerebellar regions and other DMN areas (e.g., mPFC and PCC/PCu; Bluhm et al., 2009; Liu et al., 2012). Zhu et al. (2012) observed increased anterior medial cortex (e.g., mPFC and ACC) functional connectivity and decreased posterior medial cortex (such as PCC/PCu) functional connectivity in the resting-state DMN of first-episode, treatment-naïve MDD patients.

In accordance with resting state findings, results under task state also have been inconsistent. Inconsistencies across different studies may be due to differences in study populations (Gotlib et al., 2004) and methodology. For instance, drug treatments for depression or anxiety may significantly alter emotional processing (Fu et al., 2004). In addition, prior studies used different experimental paradigms, which may assess different time courses of emotional processing (Tamietto and de Gelder, 2010; Shi et al., 2013). Grimm et al. (2011) found that during performance of a self-related task, MDD patients showed decreased deactivation in anterior medial cortical regions and increased deactivation in posterior medial cortical regions of the DMN. During emotional tasks, MDD patients showed significantly decreased deactivation in the DMN, and this deactivation magnitude correlated with depression severity and feelings of hopelessness (Grimm et al., 2009). However, Sheline et al. (2009) found that depressed patients did not show DMN deactivation during negative picture processing. Thus, it remains unclear whether people with MDD show DMN alterations under task state.

Human facial expressions are powerful social environmental cues used to signify the attitudes and emotions of others. Biased processing of emotional faces may lead to social deficiencies, such as impaired social skills and poor interpersonal relationships, which can contribute to the development of depression (Beevers, 2005). Researchers have been studied how the brain processes images of emotional faces in patients with MDD to explore MDD pathophysiology. Two ways exist in the emotional processing: explicit processing and implicit processing. As to the emotional faces processing, explicit tasks usually involves judging the strength or type of the facial emotion (Surguladze et al., 2005), while implicit tasks distract participants’ attention with a non-emotional task or presenting the emotional faces for a very short time with masking (Esteves and Ohman, 1993; Vuilleumier et al., 2001; Anderson et al., 2003). The implicit processing occurs in the early stages of attention and cognition, thus may reflect more social and evolutionary significance of emotion compared to explicit processing. Some studies have shown that people with depression have a higher threshold of information processing; therefore, masked faces may not arouse a significant reaction in them (Fusar-Poli et al., 2009). Distraction task has several advantages, which include ensuring effective sensory input channels and separating the impact of attention from being a confounding factor in emotional processing in depression (Phillips et al., 2010). Therefore, we used a gender judgment task to measure emotional processing mechanisms in depressed patients.

Almost all of the face perception related brain regions have been associated with MDD pathophysiology (Mayberg, 1997; Phillips et al., 2003). In a meta-analysis of studies of emotional faces processing in MDD, Stuhrmann et al. (2011) found that depressed patients had emotional processing network aberrations and mood-congruent processing bias. When processing emotional faces implicitly, depressed patients have been reported to show decreased activation in the PFC and hippocampus (Sheline et al., 2001; Davidson et al., 2003). It has been postulated that depression may be caused by failures of cognitive processing and emotional regulation systems (Mayberg, 1997). Supporting this view, depressed people have been observed to exhibit a failure to down-regulate activities normally (Sheline et al., 2009). Recent studies have demonstrated that maladaptive emotion regulation strategies such as rumination were associated with DMN hyperconnectivity during resting state or self-focused task (Sheline et al., 2010; Berman et al., 2011; Hamilton et al., 2011). During external-focused task or cognitive demanding task, rumination interfered with adaptive switching to goal-oriented networks (Jacobs et al., 2014; Nixon et al., 2014; Bartova et al., 2015; Belleau et al., 2015).

The limitations of previous studies lies in that: firstly, most of the task-state studies focused on activations related with cognitive and/or emotional functions, which seldom reported results of deactivations; secondly, most studies on the DMN of MDD patients were resting state studies, those few research which explored DMN alterations under task used explicit paradigms and showed inconsistent results. Moreover, the relationship between the task related network and DMN needs to be clarified. Therefore, the purpose of the current study was to address prior discrepancies in the literature and to investigate the aberrant brain network patterns of depressed patients performing an implicit emotion processing task. Only first-episode, treatment-naive young adults with MDD were enrolled in the MDD group to eliminate the effects of confounding factors such as comorbidities, duration of disease, and drugs. The implicit task allowed us to explore how MDD patients perceive emotional faces in early time courses. We compared emotional faces processing-related network and DMN patterns in MDD patients versus healthy controls. We predicted that depressed patients would show DMN alterations during the emotional faces processing task.

## Materials and Methods

### Participants

A group of 32 outpatients with MDD (12 males, 20 females) who had experienced their first depressive episode recently were recruited from the Second Xiangya Hospital of Central South University. Diagnosis of MDD was made independently by two trained psychiatrists using the Structured Clinical Interview of the DSM-IV (SCID). The MDD patients included in the study had never been diagnosed with another current Axis I disorder and had not ever been treated with psychiatric medication at the time of the fMRI recording. A group of 36 healthy controls (17 males, 19 females) were recruited through advertisements at several colleges in the city of Changsha (Hunan Province, China). The two groups were matched based upon age and educational status. The selected participants were screened for neuropsychiatric disorders by two experienced psychiatrists with the SCID, Non-Patient Edition (SCID-NP). Participants were excluded if they met any of the following criteria: any current or previous psychiatric disorder; family history of a psychiatric disorder; previous head trauma with loss of consciousness; alcohol or substance abuse history; a chronic neurological disorder; severe or acute medical condition that impacts cognitive function; contraindication to fMRI.

All of the enrolled participants had normal or corrected-to-normal vision and all were right-handed according to the Edinburgh Handedness Inventory (Oldfield, 1971). Depression severity was evaluated by the Chinese version of the CES-D (Center for Epidemiologic Studies Depression Scale), a 20-item self-report instrument with a high degree of reliability and validity designed to assess depressive symptoms in the general population (Radloff, 1977). All participants completed the CERQ (Cognitive Emotion Regulation Questionnaire; Garnefski et al., 2002), which assesses what cognitive emotion regulation strategies one uses to deal with the experience of negative events or situations. The Chinese version of the CERQ has been demonstrated to have good reliability and validity (Zhu et al., 2008).

This study was approved by the Ethics Committee of the Second Xiangya Hospital of Central South University. All participants were informed about the purpose of the study and gave written informed consent.

### Experimental Paradigm and Procedure

A set of 75 photographs of emotional faces (15 each of sad, angry, fear, happy, and neutral faces) were selected randomly from the Chinese Facial Affective Picture System (Bai et al., 2005). All of the photographs were consistent in size, brightness, and emotional intensity.

**FIGURE 1:**
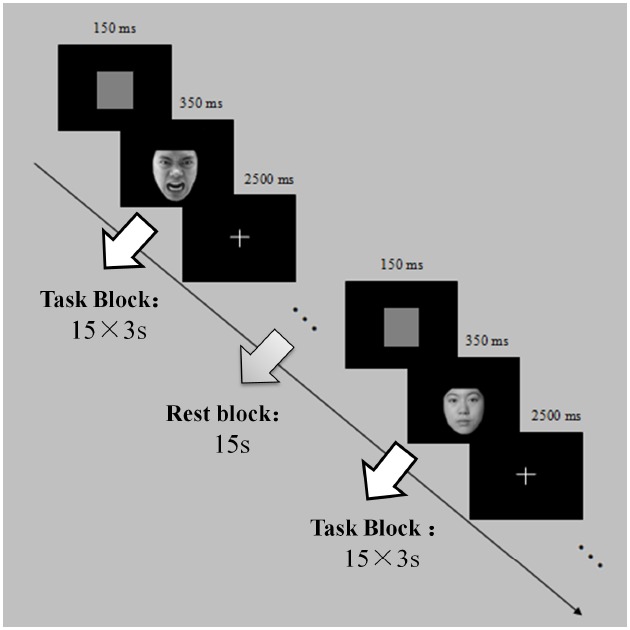
**Schematic of experimental sequence.** In each of two scanning runs, participants were presented with alternating task (45 s) and rest (15 s) blocks (five of each). Each task block had 15 trials. Each trial consisted of the serial presentation of a gray square forward mask (150 ms), an emotional face image target stimulus (350 ms), and a central fixation cross (2500 ms).

Implicit processing of emotional faces was assessed during administration of a gender judgment task. The experiment was conducted in a block design. To ensure sufficient trials for further statistical analyses, two equal sessions with random order were performed. Each session consisted of five blocks. Participants were encouraged to take a break inside the scanner after the first session. In each session, participants were presented with alternating task (45 s) and rest (15 s) blocks (five of each). A central fixation cross was presented on the screen during the rest block. Each task block had 15 trials. Each trial consisted of the serial presentation of a gray square forward mask (150 ms), an emotional face image target stimulus (350 ms), and a central fixation cross (2500 ms; See Figure 1). Participants were asked to determine the gender of the people in the photographs as soon as they saw each target stimulus by pressing response buttons with either the left or right index finger, with left versus right designation counterbalanced across participants. The whole experiment lasted for 600 s. An E-Prime 1.1 computer program (Psychology Software Tools, Pittsburg, PA, USA) controlled the experimental protocol. Participants completed the experimental task inside an MRI scanner. Before entering the scanner, each participant read detailed information about the experiment and completed practice trials to ensure they were familiarized with the task. The photographs used for practice were not the ones used in the formal experiment.

### MRI Data Acquisition

Magnetic resonance images were acquired on a 1.5-T scanner (Siemens Magnetom Symphony Scanner) with a standard head coil. Earplugs and foam pads were used to reduce noise and decrease head motion, respectively. Functional imaging data were acquired axially in an interleaved slice order using a T2* weighted gradient echo sequence parallel to the line of the anterior-posterior commissure: repetition time/echo time = 3000/40 ms, acquisition matrix = 128 × 128, voxel size = 3.8 mm × 3.8 mm × 4.0 mm, slice thickness = 4 mm, no gap, 30 slices, flip angle = 90°, field of view = 240 × 240 mm. The first four volumes of each run were discarded to allow time for the magnetization to reach a steady state. High resolution T1-weighted 3D images were also acquired: repetition time/echo time = 1900/192 ms, acquisition matrix = 256 × 256, voxel size = 1 mm × 1 mm × 1 mm, slice thickness = 1 mm, no gap, flip angle = 15°, field of view = 250 × 250 mm.

### fMRI Data Preprocessing

Imaging data were analyzed in SPM8 software^1^. Functional images were corrected for slice-timing, realigned to the first scan from each session, spatially normalized into a standard stereotaxic space at 2 × 2 × 2 mm^3^ based on the Montreal Neurological Institute (MNI) template, and then smoothed with an 8-mm full width at half-maximum Gaussian kernel.

### General Linear Model

Individual participants’ responses were modeled using a general linear model (GLM), which employed a design matrix for separate experimental conditions. To evaluate brain activation in gender judgment task, a random-effect analysis was performed that compared the task to the resting condition. The resting period was modeled as the control condition. The six motion parameters calculated during realignment were included as the regressors in the GLM. For the first level analysis, individual statistical parametric maps were calculated to elucidate the effects of implicit emotional faces processing, which resulted in emotional versus control t-contrast images for each participant in both groups. The t-contrast images were entered into second-level analysis and one-sample *t*-tests were performed to detect both activations and deactivations at the group level. Deactivation was calculated by comparing resting condition to task condition. To evaluate differences between MDD patients and controls, two sample *t*-tests were performed. Thresholds were set at uncorrected *p* < 0.001 for the first level analysis. For the second-level analysis, thresholds were set at voxel-level uncorrected *p* < 0.001 and cluster-level family-wise error (FWE)-corrected *p* < 0.05.

### Independent Component Analysis

Spatial independent component analysis (ICA) was conducted for all participants using GIFT (Group ICA of fMRI Toolbox) software^2^. Functional data of resting period was excluded in ICA analysis for the purpose of extracting DMN component under task condition. First, the optimal number of independent components (ICs) was estimated by the minimum description length criteria (Li et al., 2007), which was 27 for the MDD group and 26 for the controls. The data obtained from each participant were decomposed into spatially separated ICs using the infomax algorithm, resulting in 27 independent spatial maps for MDD patients and 26 independent spatial maps for the controls. The corresponding components for each participant were then calculated via a back reconstruction step. A DMN template derived from previous study (Laird et al., 2009) was used to select the greatest best-fit component for each participant. The ICs were sorted using a multiple spatial regression of the template with the ICs, and the one with highest regression coefficient was identified as the best-fit component representing the DMN. After the DMN components were extracted from all participants and gathered by group, one sample *t*-tests were applied to produce the group mean DMN, thresholded at voxel-level uncorrected *p* < 0.001 and cluster-level FWE-corrected *p* < 0.05. Subsequently, two-sample *t*-tests were used to compare the DMN components between the two groups, thresholded at voxel-level uncorrected *p* < 0.005 and cluster-level FWE-corrected *p* < 0.05. Relatively loose voxel-level *p* values were chosen to make sure more DMN areas with high functional connectivity were included.

To examine the association of DMN alterations with the use of cognitive emotion regulation strategies, voxel-based correlational analyses were performed between DMN components and CERQ-maladaptive scores for the MDD group. The results were constrained within the mask of the DMN regions obtained from two-sample *t*-test showing between-group differences. Thresholds were set at *p* < 0.05 with a minimum cluster size of 15, which corresponded to AlphaSim corrected *p* < 0.001.

## Results

### Behavioral Results

The fMRI data from three MDD patients and three healthy controls were excluded from the final data analysis due to excessive head motion. All the participants showed above 90% of correctness during the gender judgment task. Thus, the statistical analysis included data for 29 MDD patients and 33 healthy controls. Table 1 presents the demographic and clinical characteristics of the two groups of participants. Participants did not differ significantly in terms of gender ratio, age, or educational level. Two sample *t*-tests revealed that MDD patients had higher CES-D (*t* = 10.89, *p* < 0.001) and CERQ-maladaptive (*t* = 3.21, *p* < 0.01) scores than controls.

**TABLE 1:** **Demographic and clinical characteristics of the MDD and control groups**.

**Characteristic**	**Control**	**MDD**	**t/*χ*^2^**	**p**
Sex (male/female)	16/17	11/18	0.70	>0.5
Age (years)	20.75 ± 1.50	20.45 ± 1.80	0.74	0.46
Education (years)	13.88 ± 0.86	13.72 ± 1.03	0.65	0.52
Mean CES-D score	37.26 ± 7.67	56.10 ± 5.73	10.89	<0.001***
Mean CERQ-maladaptive score	43.71 ± 5.15	48.93 ± 7.36	3.21	0.002**

**p < 0.01, ***p < 0.001.

### GLM Results

**FIGURE 2:**
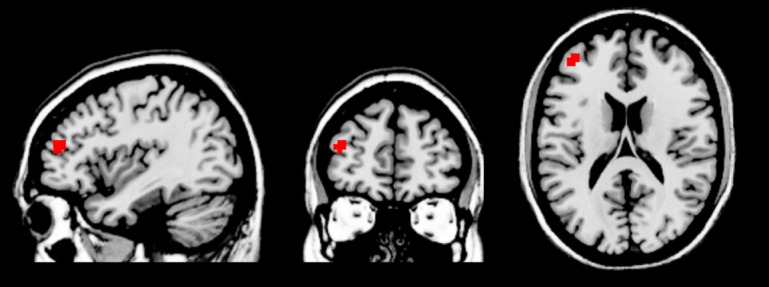
**Increased left middle frontal gyrus (two clusters, peak coordinates: x = –39, y = 48, z = 18) activation in the MDD group compared to controls during the gender judgment task**.

In the control group, the gender judgment task activated brain areas including the bilateral cuneus, frontal cortex, and superior parietal lobule, and deactivated brain areas including the bilateral superior parietal gyrus, superior frontal gyrus, ACC, and superior temporal gyrus (voxel-level uncorrected *p* < 0.001, cluster-level FWE-corrected *p* < 0.05). In the MDD group, the gender judgment task activated the bilateral cuneus and frontal cortex, and deactivated the bilateral parietal lobe, mPFC, ACC, and superior temporal gyrus (voxel-level uncorrected *p* < 0.001, cluster-level FWE-corrected *p* < 0.05). Comparison of two groups showed increased activation of left middle frontal gyrus in MDD patients during the gender judgment task (see Figure 2).

### ICA Results

**FIGURE 3:**
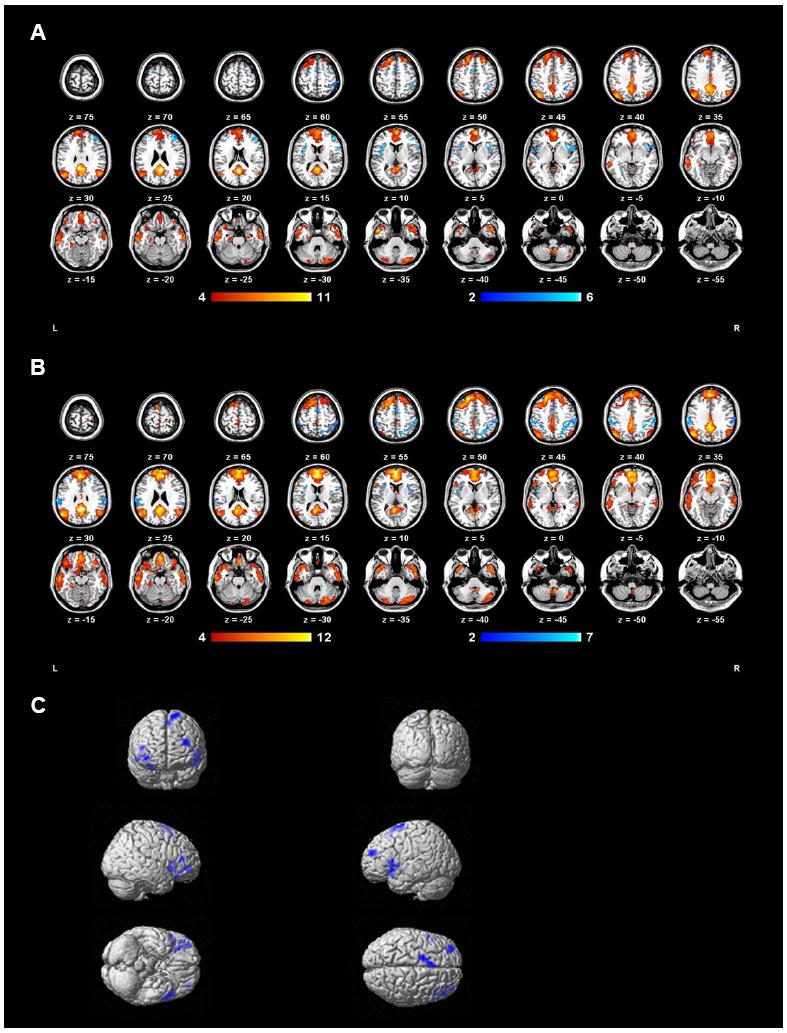
**Group DMN extracted by ICA of MDD group (A) and control group (B) subjects. (C)** DMN regions with decreased functional connectivity in the MDD group compared to controls during the gender judgment task are shown in blue.

**FIGURE 4:**
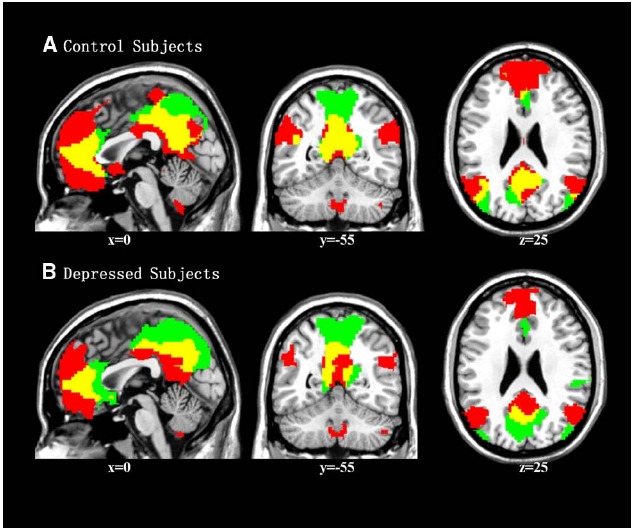
**Overlapping regions (in yellow) of the task-negative network (in green) and DMN (in red) in the control group (A) and MDD group (B)**.

One-sample *t*-tests revealed the respective spatial pattern of the DMN in the MDD patients and healthy controls (voxel-level uncorrected *p* < 0.001, cluster-level FWE-corrected *p* < 0.05; Figures 3A,B). Compared with the control group, the MDD group showed decreased functional connectivity in the bilateral PFC (voxel-level uncorrected *p* < 0.005, cluster-level FWE-corrected *p* < 0.05; see Table 2 and Figure 3C for details). We also found that the task-negative network and DMN had large areas of overlap in both groups, including the mPFC, ventral ACC, and PCu (Figure 4). Correlational analysis indicated that functional connectivity of the right inferior frontal gyrus and middle frontal gyrus correlated negatively with CERQ-maladaptive scale scores in the MDD group (*p* < 0.05, cluster size > 15, corresponding to AlphaSim corrected *p* < 0.001; see Table 3 for details).

**TABLE 2:** **DMN regions with decreased functional connectivity in the MDD group compared to controls**.

**Brain region(s)**	**Hemisphere**	**voxels**	***t***	**MNI coordinates**	**BA**
Superior frontal gyrus	Left	102	5.11	–9 12 69	6
Inferior frontal gyrus/ Superior temporal gyrus	Left	106	4.66	–45 18 9	44/22
Middle frontal gyrus	Left	61	4.60	–36 51 21	9
Inferior frontal gyrus/ Orbitofrontal gyrus	Right	70	4.30	39 21 6	45
Middle frontal gyrus/ Inferior frontal gyrus	Right	65	3.72	36 57 –6	10/47

BA, Brodmann area; Voxel-level uncorrected p < 0.005 and cluster-level FWE-corrected p < 0.05.

**TABLE 3:** **DMN regions correlated with CERQ-maladaptive scores in the MDD group**.

**Brain regions**	**Hemisphere**	**voxels**	***r***	**MNI coordinates**	**BA**
Inferior frontal gyrus	Right	16	–0.79	48 27 0	
Middle frontal gyrus	Right	17	–0.65	36 48 –6	

BA, Brodmann area; p < 0.05, cluster size > 15, corresponding to AlphaSim corrected p < 0.001.

## Discussion

In this study, we demonstrated DMN alterations during implicit emotional faces processing in first-episode, treatment-naive MDD patients. The issue of mixed comorbidity was avoided by exclusion of subjects with any Axis I comorbidities, creating an ostensibly pure MDD sample. The advantage of this study lies in that we first use ICA to detect DMN alterations under task state by using implicit emotional processing paradigm. Compared to controls, MDD patients displayed decreased mPFC functional connectivity during a gender judgment task. Scores on the maladaptive CERQ scales correlated negatively with inferior and middle frontal gyrus functional connectivity in the MDD group.

DMN activity has been shown to correlate with task-negative network activity, but to anti-correlate with task-positive network activity in healthy subjects (Fox et al., 2005). Zhou et al. (2010) found that first-episode, treatment-naïve MDD patients had increased resting-state functional connectivity in the PCC and mPFC, and their task-negative networks were anti-correlated with task-positive networks. Examining the effect of gender judgment task performance on DMN connectivity, Li et al. (2012) found large overlaps between task-induced deactivation regions and the DMN, consistent with our findings. Although the DMN regions showed deactivation during task performance, the effective connectivity within the DMN was enhanced (Li et al., 2012). This intrinsic excitability suggests that the DMN may play an important role in facilitating the switch from resting state to task state (Hampson et al., 2006). According to the theory of resource rearrangement, committing to a task results in a rearrangement of the brain resources, such as deactivation of some brain areas (Gusnard and Raichle, 2001; Raichle et al., 2001). That is, brain resources used for internal information processing in a resting state might be devoted to task-related processing, reflected by task-related activation in certain brain areas.

Our finding of less functional connectivity in the mPFC of MDD subjects during task blocks, relative to controls, is consistent with the notion that depressed people may have dysfunctions in assigning brain resources during the switch from rest to task-related activities. In the healthy brain, the DMN exhibits decreased activation during task performance, enabling a shift of resources to performance of the task (Buckner et al., 2008). Meanwhile, cognitive load imposition may shift the internal connectivity mode of the DMN anteriorly (e.g., from the PCC to the mPFC; Esposito et al., 2009). The intrinsic excitability of the mPFC suggests that it may play a major role in assigning the brain resources during the switch from resting state to task state. Using dynamic causal modeling, Li et al. (2012) found enhanced effective connectivity within the DMN of healthy subjects, especially to and from the mPFC, during a gender identification task.

In a prior study of first-episode, treatment-naive MDD patients, Zhu et al. (2012) observed increased mPFC functional connectivity under resting state, which may be related to self-referential processing. Accordingly, decreased mPFC functional connectivity in MDD patients *during task performance* is consistent with the notion that depressed people may experience less self-referential processing when they are involved in task-related activities. In a seed-based connectivity study, increased connectivity in mPFC was found in MDD during resting state, but not during task engagement (Berman et al., 2011), which was consistent with our findings. There is evidence suggesting that the DMN may engage in task performance monitoring, rather than being suppressed, during task performance (Hampson et al., 2006; Newton et al., 2011). If so, then the decreased DMN functional connectivity in MDD patients during task performance may reflect impaired internal monitoring during emotional processing. Li et al. (2013) found that the abnormal functional connectivity within the anterior subnetwork of the DMN observed in MDD patients before treatment persisted after treatment. Our results with a treatment-naïve sample support the notion that an aberrant anterior subnetwork of the DMN could be an important biomarker of depression.

Emotion regulation style is considered an influential factor in the development of depression (Garnefski et al., 2003). After experiencing negative life events, emotional responses are regulated by cognitive responses. However, maladaptive regulation strategies may hinder the development of social skills, leading to impaired interpersonal relationships and mental health. Maladaptive cognitive emotion regulation strategies, such as self-blame, rumination, and catastrophizing, correlate with depressive symptoms (Jermann et al., 2006; Lei et al., 2014). Individuals with higher CERQ subscale scores suggest that they are more likely to use the corresponding cognitive strategies. Hence, the negative correlation between maladaptive CERQ scale scores and prefrontal gyrus functional connectivity in the MDD group observed in the present study suggests that decreased prefrontal functional connectivity in depressed patients may be related to their deficiencies in emotional regulation and cognitive control. Recent studies have demonstrated that maladaptive cognitive emotion regulation strategies such as rumination were associated with DMN hyperconnectivity during resting state or self-focused task (Berman et al., 2011; Hamilton et al., 2011). During external task or cognitive demanding task, MDD patients showed difficulties in relocating neural resources to goal-oriented networks, and rumination partially mediated the relationship between attentional dyscontrol and depressive symptoms (Demeyer et al., 2012; Lemogne et al., 2012; Nixon et al., 2014; Belleau et al., 2015). It was suggested that engaging in a task could disrupt self-referential processing and improving mood (Nolen-Hoeksema et al., 2008). As increasing evidence showed self-referential processing played a major role in the development, course and treatment of MDD (Nejad et al., 2013), our findings may provide clinical implications for MDD patients in terms of emotional regulation. The limitation of this study lies in the group ICA approaches employed because there is no standard method for choosing the optimal number of components (Zuo et al., 2010), and results may vary with different concatenation orders of the data (Zhang et al., 2010). In addition, the functional mechanisms of the DMN remain unresolved. Therefore, full interpretation on the current results will require future research.

In conclusion, this study demonstrated that first-episode, treatment-naive young adults with MDD exhibited DMN alterations during emotional faces processing, as shown by lesser functional connectivity of the mPFC than controls bilaterally. Additionally, functional connectivity of the mPFC correlated negatively with maladaptive cognitive emotion regulation strategy habits. These findings further indicate that patients with MDD have impaired internal monitoring ability and cognitive emotion regulation, and are consistent with the notion that these characteristics may contribute to the development of the disease.

### Conflict of Interest Statement

The authors declare that the research was conducted in the absence of any commercial or financial relationships that could be construed as a potential conflict of interest.

## Footnotes

1 www.fil.ion.ucl.ac.uk/spm

2 http://icatb.sourceforge.net
